# Revisiting the CompCars Dataset for Hierarchical Car Classification: New Annotations, Experiments, and Results

**DOI:** 10.3390/s21020596

**Published:** 2021-01-15

**Authors:** Marco Buzzelli, Luca Segantin

**Affiliations:** Department of Informatics Systems and Communication, University of Milano-Bicocca, 20126 Milano, Italy; l.segantin@campus.unimib.it

**Keywords:** hierarchical car classification, car detection, car dataset, CompCars

## Abstract

We address the task of classifying car images at multiple levels of detail, ranging from the top-level car type, down to the specific car make, model, and year. We analyze existing datasets for car classification, and identify the CompCars as an excellent starting point for our task. We show that convolutional neural networks achieve an accuracy above 90% on the finest-level classification task. This high performance, however, is scarcely representative of real-world situations, as it is evaluated on a biased training/test split. In this work, we revisit the CompCars dataset by first defining a new training/test split, which better represents real-world scenarios by setting a more realistic baseline at 61% accuracy on the new test set. We also propagate the existing (but limited) type-level annotation to the entire dataset, and we finally provide a car-tight bounding box for each image, automatically defined through an ad hoc car detector. To evaluate this revisited dataset, we design and implement three different approaches to car classification, two of which exploit the hierarchical nature of car annotations. Our experiments show that higher-level classification in terms of car type positively impacts classification at a finer grain, now reaching 70% accuracy. The achieved performance constitutes a baseline benchmark for future research, and our enriched set of annotations is made available for public download.

## 1. Introduction

Every day, a very large number of vehicles passes through the streets of our cities and towns. In fact, even though the car sales market volume is far from its 2007 peak [1], statistics from 2016 [2] state that in Italy alone there are 625 passenger cars for every 1000 inhabitants, without considering trucks or motorbikes. It should not come as a surprise, then, that the idea of detecting and classifying these vehicles can be useful for a wide range of applications, from security to commercial use. For example, private parking lots usually employ camera-based sensors that detect each vehicle’s license plate at the entrance, and store the information for a later matching with the parking receipt at the exit. While this method is useful for tracking and managing the traffic passing through, a thief could enter the parking lot with a cheap stolen car, swap the license plate with one of the parked cars, steal it, and leave the place unhindered. By knowing the make and model associated with each license plate, a cross-check could be made at the exit to avoid these situations and increase the security of the customers’ property. For commercial applications, then, vehicle detection and recognition have been successfully employed in advertising campaigns by media agencies [3], where interactive billboards are equipped with in-loco sensors that recognize the incoming vehicle and present a user-targeted ad. A recent survey on vehicle classification [4] highlighted the importance of intelligent traffic monitoring systems to mitigate traffic congestion. The authors pointed out that automatically collecting information on number of vehicles, type, and speed, can be used to fine-tune traffic analysis, and thus to better exploit the roadway systems, improve the safety of transportation, and predict future transportation needs.

These scenarios offer a compelling case for the identification of a car’s make and model. In light of the efficacy of machine-learning approaches to detection and classification [5], then, the definition of high-quality datasets is of paramount importance. We argue that an effective dataset should meet at least the following criteria:

- High cardinality. One key role in the advent of deep learning has been the availability of large collections of annotated data [6], a notable example being the ImageNet dataset [7].
- Annotation richness. Redmon et al. [8] partially explained the gap in performance between object detection and the more mature image classification, with the availability of hierarchical-class annotations for the latter category (i.e., every image is associated with more than one label, at different levels of abstraction).
- Challenging task. As research advances, new and more challenging datasets have to be constantly introduced [9,10]. Particular care should be also put in the definition of the training and test splits, in order to prevent unwanted biases [11].

The collection of any new dataset is, however, a particularly resource-consuming process. Furthermore, recent regulations that are rightfully aimed at defending the privacy of individuals (e.g., the European General Data Protection Regulation, or GDPR), had the unavoidable effect of limiting the possibilities of gathering and publicly distributing new collections of image data [12].

Driven by these observations, we conduct an overview of existing datasets for the detection and classification of cars, with special attention to the labeling of their “make” and “model”. We highlight the characteristics of such datasets, and eventually determine the CompCars [13] to be a suitable candidate for revisiting and enhancing its annotations. We thus provide the following contributions:

1. We define a new challenging training/test split for the CompCars dataset.
  Differently from a random split, this solution prevents near-duplicates between training and test set, resulting in a more representative test set.
2. We propagate the “type”-level annotations to the entire dataset.
  This highest-level label is originally present only for some of the vehicles in the dataset, thus limiting the possibility of exploiting hierarchical classification.
3. We provide a car-tight bounding box for each image.
  By treating the problem as a detection task, we provide a set of automatic bounding boxes that are shown to significantly improve classification performance.
4. We define and train several solutions for the task of car detection and hierarchical classification over the revisited CompCars dataset.
  These serve as baseline benchmarks for future work by other researchers.

Thanks to the above contributions, the scientific community can further exploit the CompCars dataset as a new challenging setup for hierarchical car classification with rich annotations. All the new data and annotations are made publicly available.

## 2. Car Classification Datasets

**Figure 1:**
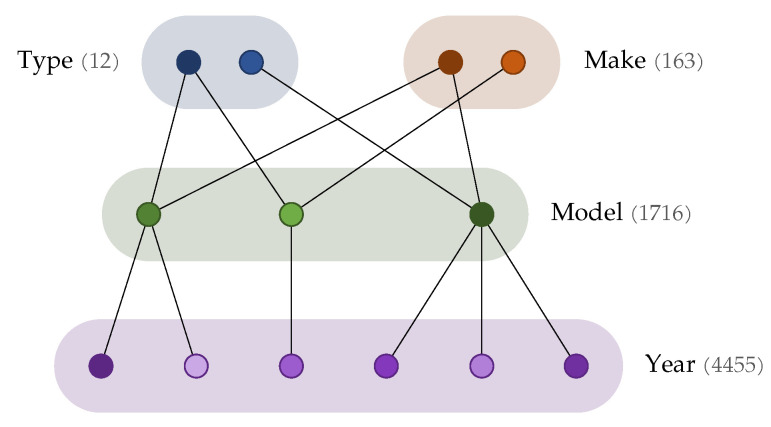
Hierarchy of labels in the CompCars dataset, comprised of a total of 136,725 images. The reported numbers identify the cardinality of the classes at each specific level.

In the following, we use the nomenclature commonly adopted by existing datasets, referring to a car’s type, make, model, and year of release. The hierarchy of these labels is visualized in Figure 1.

The type-level annotation describes a brand-independent identification of the vehicle’s purpose and line of design (e.g., Sedan, Convertible, etc.). The make-level annotation refers to the producing brand (e.g., Audi, BMW, etc.). Since a single brand typically releases vehicles of different types, these labels are independent, and “make” is commonly used in retrospective analysis instead of being the direct target for classification. The model-level annotation describes the specific series (e.g., Audi Q3, Audi Q5, etc.), and it is directly associated with a specific make. Finally, the year-level annotation identifies different releases of a specific make and model (e.g., BMW M3 2010, BMW M3 2014, etc.). While in some cases the differences between years are quite radical, others introduce only slight and subtle variations from one year to another, making this the most challenging level of classification. The path of labels composed of make–model–year is referred to as a MMY triplet, which by extension also refers to the deepest level of annotation (year). The cardinalities reported in figure refer to CompCars, which, as described in the following, is selected as the reference dataset for the purpose of this paper.

Some important characteristics for the choice of a car classification dataset are the number of images for each class, the exhaustiveness of the car models, and the level of detail of the labels. A total of three datasets have been considered: the CompCars [13], the Stanford [14], and the VMMR (Vehicle Make and Model Recognition) [15]. Table 1 shows an overview of their characteristics.

The **CompCars** (Comprehensive Cars) dataset [13] from 2015 contains data from two scenarios, respectively called “surveillance-nature” and “web-nature”. The surveillance-nature set is composed of 50,000 images of frontal-view cars, associated with bounding box, car model, and color. The web-nature set contains a total of 136,725 images, where each car model is labeled with its car MMY triplet (considered at three different levels of detail, there are 163 makes, 1716 models, and 4455 models from different years) plus other attributes, notably the car type label. This dataset presents some classes with low cardinality and groups of similar images. However, the hierarchy of annotation labels makes it possible to address the classification problem at different levels of detail (hierarchical classification [16]), as described in Section 4. In addition to hierarchical labels, the CompCars dataset is also annotated in terms of viewpoint (frontal, rear, side, frontal-side, rear-side), and model-specific attributes (maximum speed, displacement, number of doors, number of seats). A set of car part images is also provided, belonging to eight categories (headlight, taillight, fog light, air intake, console, steering wheel, dashboard, gear lever).

The **Stanford** dataset [14] from 2013 contains 16,185 images of 196 classes at the lowest level of make–model–year (MMY). The data consist of 8144 training images and 8041 testing images, where each class has been roughly divided in a 50-50 split. Some of the MMY triplets have a concept similar to the car type embedded within the label as well. Generally speaking, the dataset has a small range of car makes and models, with a limited number of images and basic labels. An additional small set of 512 images called the BMW-10 Stanford dataset is available with 10 models from different years from the BMW car make. The **VMMR** (Vehicle Make and Model Recognition) dataset [15] from 2017 contains 9170 classes consisting of 291,752 images (not split in train and test set), covering car makes, models and years between 1950 and 2016. The dataset contains generally smaller images, compared to the previous two. These images were taken from multiple view angles to try to account for various scenarios that could be encountered in real-life situations. There are no additional labels provided with the dataset, thus limiting the possible approaches for classification. Furthermore, the classes distribution is not uniform, with some classes having a very small number of images. More recently, the **CarVideos** dataset [17] has been introduced for fine-grained car classification in video sequences, therefore representing a different scope and problem formulation. It is composed of ten car classes at the MMY level, with 129 videos for a total of 1,529,846 frames. Despite the low number of classes, the CarVideos dataset represents an important initial step for the domain of vehicle classification in video sequences.

By considering dataset cardinality and label detail, the CompCars dataset has been identified as a valuable resource for car classification. It is, however, characterized by a number of shortcomings, which are further explored and addressed in Section 3.

### Existing Literature on the CompCars Dataset

Up to recent years, different works have used the CompCars dataset as a benchmark for car classification. In 2019, Ma et al. [18] developed a so-called Channel Max Pooling (CMP) scheme to improve the generalization ability of existing neural networks for image classification while reducing the computational burden. The authors focused on a 52,083-images subset of the CompCars dataset, and initially trained a plain DenseNet-161 [19], VGG16 [20], and ResNet-152 [21], reaching about 97% accuracy. The high performance obtained with off-the-shelf neural networks suggest that the problem is exceedingly simple, either due to the nature of the task in general, or to a bias in the specific dataset. Coherently, the authors experienced diminishing returns when introducing their CMP scheme, obtaining only slightly superior accuracy (while in return drastically reducing the neural model weight).

Fukui et al. [22] presented Attention Branch Network (ABN): a general purpose technique to embed response-based visual explanation into existing neural networks. Experimenting with CompCars, the authors first trained a vanilla VGG16 [20] and a ResNet-101 [21] network with Stochastic Gradient Descent, and obtained respectively 85.9% and 90.2% accuracy at the car model level. Thanks to their ABN, they then reported a significant increase in accuracy reaching, respectively, 90.7% and 97.1%. Despite the different results, this work also depicted a reality where the classification task of the CompCars dataset in its original form can be easily solved. Given the intrinsic complexity of the task (fine-grained classification with a large number of classes), a likely explanation can be found in the presence of a bias in the current training/test split, with near-duplicates shared between the two parts. This observation is further explored and addressed in Section 3.1.

Fomin et al. [23] presented a system for car recognition based on cascaded classifiers: they first identify the car orientation with a VGG-16 neural network [20], and subsequently select a different car-model classifier based on the found orientation, using an Inception-ResNet-v3 [24]. Following a similar concept, we will also experiment with a cascade of classifiers, although based on different abstractions: car type and car year of release. Furthermore, in their paper, the authors assumed having multiple instances of the same car as input, and returning a classification output by consensus, while we focus on a single-image input.

Partially related to the task of car classification, vehicle re-identification consists of recognizing a specific instance across a set of alternatives with similar appearance, when license plate recognition is not reliable or not available. To this extent, in 2020, Qian et al. [25] developed an attribute-aware re-identification network that exploits information relative to the car model and type to build a rich and effective feature representation. Given the different nature of the problem, they focus on re-identification datasets VehicleID [26] and VeRi [27], while the CompCars dataset is only used as pretraining set for a baseline based on the GoogLeNet architecture [28].

## 3. Revisiting the CompCars Dataset

This section describes how we revisited the CompCars dataset in terms of annotation detail and quality. We first show that a random training/test split introduces a bias in the learning process, so we produce an alternative, unbiased, split. Then, we note that the highest-level “type” annotation is only assigned to some of the car models, limiting the possibilities of hierarchical classification, so we design a strategy to propagate the type label to the entire dataset. Finally, we observe that images in the CompCars dataset often contain multiple distracting elements, so we train and run a car detector to generate car-tight bounding boxes.

The resulting set of enhanced annotations is made available for public download [29].

### 3.1. Defining an Unbiased Training/Test Split

The CompCars dataset is released with an official training/test split for car classification in the web-nature scenario. However, out of the total 136,725 images and 1716 models, only 30,955 images belonging to 431 models are part of the official setup. Furthermore, this split is designed for mild-grained classification, where different years of the same model are merged into one.

To fully exploit the entire dataset for classification, we initially divide it with a 70/30 random split on each one of the 4455 MMY classes. For reference, training a ResNet-50 neural network [21] without any data augmentation, and, without cropping the image to the actual vehicle, achieves 90% accuracy on the test set. Investigating this result reveals that this value of accuracy is not really representative of the effectiveness of the classifier when used with images taken in a more “realistic” context. In fact, a sample-based overview showed that, while images from a given class usually present a wide variety of angles, contexts and car body colors, it is not that rare to find classes with pictures that are extremely similar one to another. By resorting to a purely random split, these images are distributed in a roughly uniform way between the two sets, making the test set too similar to the train set.

In order to perform a more meaningful split where train and test sets are less trivially correlated, the images of the dataset can be considered as N-dimensional points in a feature space, and the training/test split can be defined by using criteria that are more appropriate than random sampling. To this extent, each image $x_{i}$ for a given class is transformed into a 512-dimensional point by performing feature extraction (*F*) with a ResNet-34 neural network:

(1) $$F:Z^{\mathrm{width}\times \mathrm{height}}\to R^{512}$$

For each image (point), the average distance *d* from all of the other *I* images of the class is calculated:

(2) $$d\left(x_{i}\right)=\frac{1}{I}\sum_{j=1,j\neq i}^{I}euclidean\_distance\left(F\left(x_{i}\right),F\left(x_{j}\right)\right)$$

These distances are then sorted by ascending order, and the top 70% is picked as the training set, while the remaining images become the test set:

(3) $$split\left(x_{i}\right)=\left\{\begin{array}{cc} “\mathrm{train}” & \mathrm{if}\;\;d\left(x_{i}\right)<percentile_{k=1}^{k=I}\left(d(x_{k}),70\right)\\ “\mathrm{test}” & \mathrm{otherwise} \end{array}\right.$$

**Figure 2:**
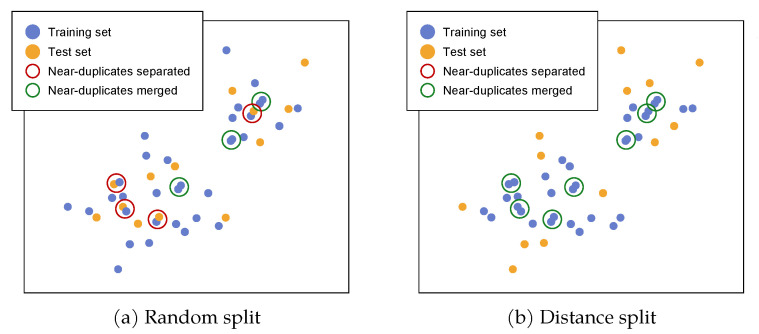
Samples of a single car class displaced in a low-dimensional space for illustration purposes. A random training/test split (**a**) might separate near duplicates between training and test set, thus creating a bias. A split based on visual distance (**b**) ensures that groups of instances that are too similar remain in the training set, resulting in a more challenging and realistic test set.

These operations are performed for every class, one class at a time. The result is visually shown in Figure 2. The final dataset is composed of 95,710 training images and 41,015 test images.

For reference, training a ResNet-50 on the split defined with this method achieves 61% accuracy on the corresponding test set, in opposition with the 90% accuracy obtained with the random split. This lower value is deemed more representative of the performance of the classifier for a scenario in-the-wild, or generally for a worst-case scenario. We argue that this renders the dataset more useful for assessment of real-world applications, and it provides a challenging setup to stimulate advances in the research. It should be noted that the provided split can be used for classification at all levels of the dataset hierarchy.

### 3.2. Propagating the Type Label for Hierarchical Classification

**Figure 3:**
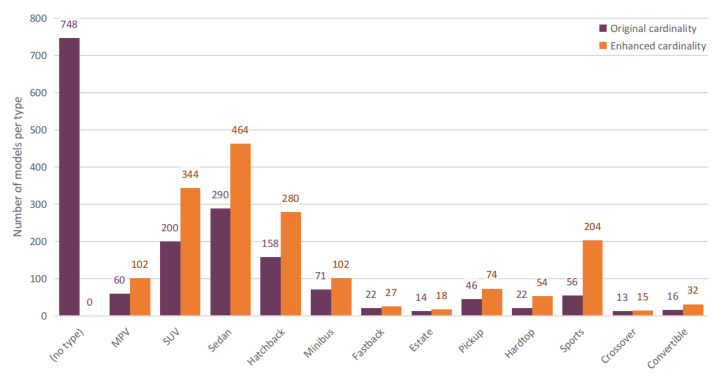
Distribution of the car type classes. Each column represents the number of car model classes belonging to a certain car type (different years are merged into one model for this representation). Dark bars refer to the original CompCars dataset, orange bars refer to the dataset revisited with our label propagation.

The CompCars dataset is divided into 12 different car types. However, only 968 car models out of 1716 are actually associated with a “type” label, as shown with the dark bars in Figure 3.

In order to potentially exploit the advantages of hierarchical classification, we set out to propagate the type-level annotation to the entire dataset, i.e., to the remaining 748 model classes (recall, from Figure 1, that each model is associated with a type). To this extent, we train a classifier on the labelled images, use it to assign labels to the unlabelled images, and finally assign labels to the models through a hierarchical consensus voting. A ResNet-50 architecture is trained on those images that are already associated with a given type label, and run on the remaining ones. A majority vote mechanism is then introduced to assign the type labels to each model: we select the most-occurring type prediction for each year, and count it as a vote towards that car model’s type prediction. The most voted car type becomes the definitive type prediction associated with the car model.

We then retrained the ResNet-50 for type classification on the entire set of car models, using the training/test split defined in the previous section. This allows us to potentially revise the type-level classes of already-labelled car, based on a global consistency across the entire dataset. The car type classifier is thus run on the test set and, by using the majority vote mechanism, the type labels for each car model are reassigned. For the vast majority of the dataset, this does not produce any changes, but, in some cases, the labels are switched to another type class: 72 models out of a total 1716 (about 4%). We manually verified such changes that are justified by the fact that the twelve car types share some common characteristics. The final type-level cardinality is shown in Figure 3 with orange bars.

### 3.3. Adding Car-Tight Bounding Boxes

The CompCars dataset depicts cars that are not tightly fit to image borders, and they are instead often embedded in a wider context. Furthermore, the 136,725 images of the web-nature scenario used in this work do not come with a bounding box annotation. For these reasons, we define an object detection pipeline to automatically assign a bounding box to each and every image. We use the resulting crop in all our subsequent experiments, and we make the coordinates available as part of our revisited dataset. However, it should be noted that these crops are not individually verified.

We train a Faster R-CNN network [30] using two possible backbone architectures: VGG16 [20] and ResNet-101 [21]. Since the images of CompCars depict real-world scenarios, many other elements are often present in the surroundings of the main vehicle, including pedestrians as well as other vehicles. We therefore train the detection network on the general-purpose PASCAL VOC 2012 dataset [9], making it aware of the existence of other object classes, and thus more robust in correctly identifying car instances. In order to assess the detection quality, and to select the most appropriate backbone, we test on the PASCAL 2007 dataset, since the test set from the 2012 version of the dataset comes with no annotations. We use a multi-task loss (that combines the classification’s cross-entropy loss and the detection’s smooth L1 loss [30] functions) as a training metric and the mAP (mean Average Precision) as a testing metric. The two backbones produce comparable performance levels in generic object detection (0.730 mAP for VGG16 and 0.765 mAP for ResNet-101), and vehicle-specific detection (respectively 0.817 and 0.830). Given the overall slight superiority of ResNet-101, we select it to produce the car bounding boxes.

**Figure 4:**
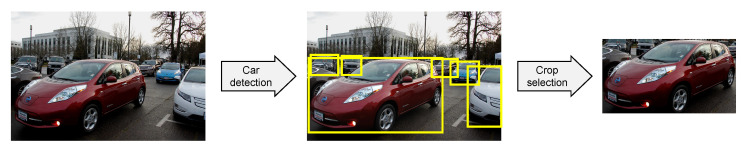
Car-tight bounding box annotation pipeline. Original image “Clean cars” by OregonDOT is licensed with CC BY 2.0.

In some cases, more than one car is detected inside of an image. Since these other cars are usually far away in the background, we define a strategy to automatically select only one element. Two different approaches have been experimented: highest confidence bounding box and biggest bounding box. An empirical analysis performed on a sample of the dataset suggests that the highest confidence method sometimes selects the wrong crop, while the biggest bounding box one looks more robust and for this reason it is the chosen one between the two. The process is visualized in Figure 4.

## 4. Car Classification

The main advantage of introducing our novel training/test split over a randomly-defined one is the guarantee that no near-duplicates are shared between training set and test set. As such, the performance of any method on the test set can be considered a reliable representation of a real-world application. One inherent disadvantage of introducing a virtually-new dataset is the initial absence of reference performance. Existing methods are in fact trained and evaluated on a different split (one potentially affected by bias, as shown in Section 3.1). To this extent, we bootstrap the comparison with future works, by defining and training three different approaches for car classification. These will serve as a reference for any further research.

**Figure 5:**
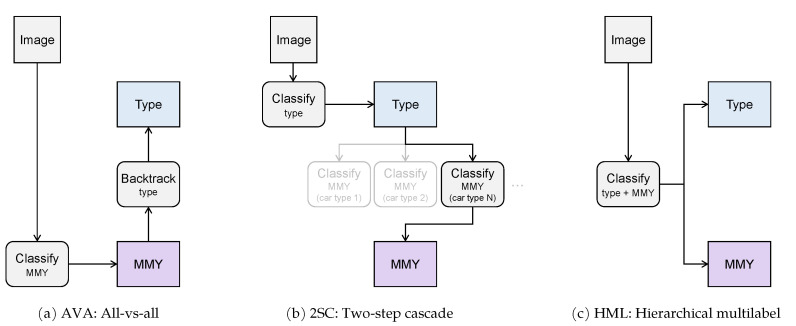
Three classification approaches devised for our revisited version of the CompCars dataset. All approaches predict the finer level of make–model–year (MMY) and the coarser level of car type.

We perform both train and inference on the crops generated by our car detection network, defined in Section 3.3. This makes it a two-stage car detection problem, composed of proposal and classification, which allows us to explore the classification stage with different approaches. We focus on convolutional neural networks in virtue of their consolidated potential in image classification. Several architectures can be considered [31] for the task, and we selected the ResNet-50 [21] due to its balance of weight and expected accuracy, and due to its widespread availability across multiple deep learning frameworks. The ResNet architecture is built on the concept of residual learning: inside each repeating block, the network is trained to learn the residual information with respect to the input of the block, instead of a novel representation without any reference. In practice, this strategy is implemented through a skip connection, or a shortcut that directly sums the block input with the block activation. One collateral advantage of introducing this specific learning strategy is the successful handling of vanishing gradients. Starting from this baseline, we then define two additional classification approaches that successfully exploit the hierarchical nature of our extended set of annotations: a two-step cascade of classifiers, and a hierarchical multilabel classifier. The three approaches are illustrated in Figure 5: the all-vs.-all approach directly predicts the finest-level class detail, the two-step cascade introduces a first high-level classifier to narrow the finest-level search domain, and the hierarchical multilabel performs a joint prediction at both levels of the hierarchy. In all cases, our goal is to exploit the dataset annotation at its finest level, i.e., to solve the classification task for 4455 year (MMY) classes. An in-depth analysis is provided in the following section.

### 4.1. Hierarchical Car Classification Approaches

**Figure 6:**
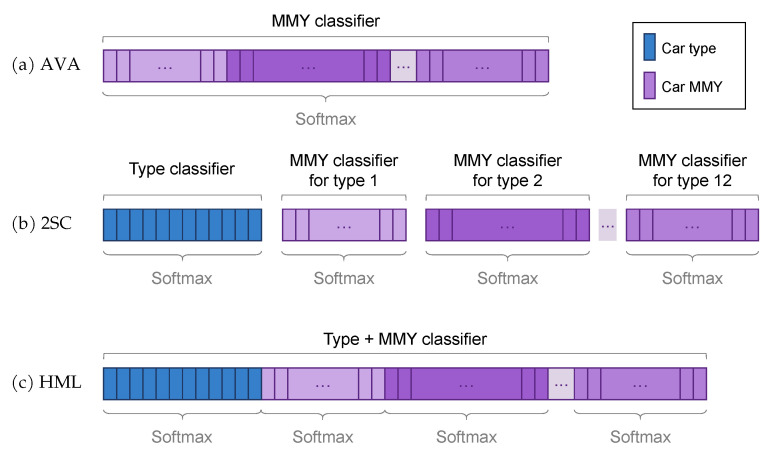
Visual representation of the classifier output for the three presented approaches. (**a**) all-vs.-all uses a unique classifier for all the finest-level make–model–year classes (MMY); (**b**) two-step cascade defines a first car-type classifier whose output points to one of 12 type-specific MMY classifiers; (**c**) hierarchical multilabel uses a single classifier to simultaneously predict type and MMY, but the backpropagation is guided by independent softmax functions for the type outputs, and for each group of type-specific MMYs.

**All-versus-all (AVA):** this basic approach considers each make–model–year triplet as a different class. For example, if a car model has five versions from different years, each of these versions becomes a stand-alone class with the label *makeX_modelY_year1,..., makeX_modelY_year5*. A single classifier can then be trained on the 4455 classes generated this way, whose output is shown in Figure 6a. The very large number of classes is expected to negatively impact the overall accuracy of such a straightforward method. This solution does not directly exploit the hierarchical nature of the dataset annotations, as it directly predicts the finest level of detail. The higher-level classes can however be inferred by backtracking the known hierarchy after the MMY prediction has been made.

**Two-step cascade (2SC):** this approach requires a two-level system of classifiers. The first level is a car type classifier that predicts which type-specific classifier forwards its input image for the final MMY classification. This approach, whose output is visualized in Figure 6b, is viable because the CompCars dataset is divided into 12-car types. This means that one first level car type classifier plus a 12 s level different MMY classifiers are everything that is needed to implement it. While this method is expected to be computationally heavier than the first one, the rationale behind it is that, by having simpler individual classifiers, their accuracy should be higher and, by combining them together, the overall accuracy of the system could surpass that of the all versus all approach. Alternatively, it is theoretically possible to devise a different two-level subsystem, where the first level is a car *make* classifier that predicts to which *make-specific* classifier forward its input image for the final classification. The problem with this approach is that it would require one classifier for the first level plus 159 different classifiers for the second level (one for each car make).

**Hierarchical multilabel (HML):** this approach proposes to carry out with a single network the same task of the Two-step cascade, speeding up the process and making it more conservative memory-wise. In a general multilabel classification problem, each image can be associated with more than one class, making the classes independent from each other. In our specific case, however, we need to define groups of mutually-exclusive classes to correctly represent the hierarchical annotation. Taking inspiration from Redmon et al. [8], the labels assigned to each image become tuples consisting of the car type and the MMY triplet. The output of the network is redesigned to contain the car type classes in the first twelve positions, and then the MMY classes ordered for each car type, 1 through 12. This is visually shown in Figure 6c.

The prediction of the car type corresponds to the highest confidence value in the first twelve positions of the output, and the car type loss is computed over these same positions for each batch. Then, for all the images in the batch, the MMY prediction is the highest confidence logit in the corresponding positions of the output of the car type ground truth label. The MMY loss is computed over these positions singularly, averaged for all the images of the batch, then summed with the car type loss, and backpropagated. At inference time, the car type interval selects in which of the following intervals to search for the highest-confidence MMY prediction. A potential advantage of the HML approach is the ability to provide an output response based on probability constraints: if the confidence of the MMY prediction is not acceptably high enough, the network could stop at the higher level and only predict the car type label, which is generally more confident in virtue of the higher abstraction.

Table 2 offers a comparison between the three methods for hierarchical car classification presented in this paper (AVA, 2SC, and HML), and existing solutions designed or adapted to the CompCars dataset, as presented in Section 2: CMP [18], ABN [22], and the method by Fomin et al. [23]. Of the latter group, only Fomin et al. exploit a version of hierarchical classification, in the form of a cascade of classifiers for car orientation and car make. We will show with experiments that properly introduce knowledge about the hierarchy of labels (as done in our 2SC) can lead to an improvement in performance (compared to the AVA approach). The main disadvantage of 2SC with respect to many other solutions is that it requires a considerable number of classifiers (one for the type, and one for make–model–year for each identified type). This translates to higher training time, larger memory occupation, and roughly double the inference time, which depending on the application might constitute a practical limitation. For completeness, we also report the architectural backbone utilized for each solution. We note, however, that all solutions can potentially be implemented on top of either of the reported neural networks.

### 4.2. Loss Functions

All of the classification approaches described so far exploit a categorical cross-entropy (CCE) loss during training, which is built on the assumption that only one class in the set of *C* classes should be predicted with high confidence by the underlying neural network (in the case of HML, this applies independently to each group of predictions):

(4) $$L_{CCE}\left(x,y\right)=-\log\left(\frac{e^{x[y]}}{\sum_{i=1}^{C}e^{x[i]}}\right)$$

with output confidence vector $x\in R^{C}$ and ground truth index y∈{1,2,…,C}. An alternative approach is to treat each class as a binary classification problem using the binary cross-entropy loss (BCE). With this method, the score for each class is considered separately from the others. First, a sigmoid function σ is applied to each logit in order to rescale it in the range [0,1]. Then, the BCE loss is computed by calculating the distance from the ground truth value: for the correct class, the loss is the distance between 1 and the logit, while, for the other classes between 0 and the logit:

(5) $$L_{BCE}\left(x,\bar{y}\right)=\sum_{i=1}^{C}\left(\bar{y}\left[i\right]\log\left(\sigma (x[i])\right)+\left(1-\bar{y}\left[i\right]\right)\log\left(1-\sigma (x[i])\right)\right)$$

with one-hot-encoded ground truth $\bar{y}={\left\{0,1\right\}}^{C}$. The difference between CCE and BCE is that the former tends to penalize correct predictions where the correct class score is not significantly higher than the other ones, while the latter (by considering the scores separately) takes into consideration that, sometimes, it can reasonable that similar-looking classes score similar results, and a correct prediction does not need to be penalized as much.

### 4.3. Introducing a Rejection Class

In our pipeline, we assume that a car-only filter is effectively implemented with the detection step described in Section 3.3. However, like with any close-set classification task, it is always possible that a car from an unknown type, make, model, or year, gets processed. It is useful to be able to intercept these scenarios and assign a “reject” class to the corresponding images.

A possible solution is applying a threshold on the confidence of the prediction. The optimization of such threshold requires the availability of a data split across MMY triplets: in this way, it is possible to determine whether a good threshold value could exist that makes the classifier recognize rejection class members and not reject too many images from the known classes. For a given classifier, we compute the F1 score obtained by rejecting instances at different confidence values, defined between 0 and 100, for both the car type and the MMY labels. For each threshold value, these two F1 scores are summed together and the highest total score identifies the optimal threshold. The F1 score is the harmonic mean between precision and recall, with its best possible value being 1 and its lowest being 0:

(6) $$F1=2\cdot \frac{Precision\cdot Recall}{Precision+Recall}$$

where

(7) $$Precision=\frac{TP}{TP+FP}\;\;\;\;\;\mathrm{and}\;\;\;\;\;Recall=\frac{TP}{TP+FN}$$

with TP being True Positives, FP False Positives, and FN False Negatives. The predictions with a higher confidence value than the threshold that are correct are considered as True Positives, while the incorrect ones are considered as False Positives. For the predictions with lower confidence than the threshold, the correct ones are False Negatives and the incorrect ones are True Negatives.

## 5. Experiments

In this section, we describe the results of different approaches to car classification, using the unbiased training and test split from our revisited version of the CompCars dataset. We train all our neural networks for the same fixed number of epochs, which was set to 30 after preliminary experiments, using Stochastic Gradient Descent with 0.001 learning rate and 0.9 momentum. We assess the performance at two opposite levels of the classification hierarchy: make–model–year (MMY, being the most challenging level with 4455 classes), and type (with only 12 classes).

### 5.1. Data Preprocessing

We process image crops resulting from the detection step described in Section 3.3. The operations performed with data augmentation are random rotations in a 20-degree range, random translations, and color jittering. The rotation and translation operations are used to improve the robustness of the classifier on pictures with various points of view and partially occluded cars. The color jittering transformations have been introduced because some classes of the dataset present the car in the same color in all of their instances. This can be a problem during training, since a certain class could be associated with a certain color and new pictures of the same car with a different painting could be misclassified based on color alone. This method encourages the network to learn to ignore the color information, limiting the problem caused by classes with cars of the same color.

### 5.2. Classification Results

Table 3 illustrates the result of hierarchical car classification using the three devised approaches: all-vs.-all, two-step cascade (in two loss variants), and hierarchical multilabel. Detailed MMY accuracy for each car type is also provided.

In the **All-vs.-all (AVA)** approach, the problem consists of 4455 car make–model–year classes, and the network is trained to predict directly to which one out of these classes the input image belongs to. After 30 total epochs, the best values registered from the neural network are 0.940 for training set accuracy, and 0.688 for test set accuracy. This result can be compared to the 0.61 accuracy described in Section 3.1, where no car detection was performed, and as such it highlights the importance of performing classification on a car-tight crop. The MMY triplet can then be mapped “backwards” in the dataset hierarchy to infer the corresponding car type label, resulting in 0.907 accuracy. Detailed performance is shown in the first column of Table 3.

This experiment was performed using categorical cross-entropy (CCE). A second training using binary cross-entropy (BCE) as described in Section 4.2 has also been performed, causing, however, a drastic decline in the accuracy of the all the classes. For this reason, the results are not reported in the table. The underwhelming results achieved when training the MMY classifiers with the BCE loss are probably caused by the fact that, in this configuration, the dataset classes often contain a very low number of examples. While the cross-entropy loss tends to have a bias towards the larger classes, the BCE loss gives the same importance to every class, and the small number of examples makes it difficult to extract a good generalization of these classes. The large number of classes could also have a particularly negative effect on BCE.

In the **Two-step cascade (2SC)** approach, the car “type” labels are used to introduce an intermediate step during the classification. In this way, it is possible to split the task into two smaller sub-tasks: the first one being the car type label (12 total) classification of the input image, and the second one being the make–model–year classification performed by one of 12 car type-specific MMY classifiers, chosen by the first level prediction.Each of these classifiers is trained to predict the car MMY triplet of only the subset of the total classes, making it an inherently-simper task. On the other hand, when the first-level car type classifier makes a wrong prediction, the image is sent to the wrong second-level car MMY classifier, making it effectively impossible to obtain a correct prediction. The detailed results are reported in the second column of Table 3, consisting of 0.907 accuracy for the car type, and 0.680 accuracy for MMY (slightly lower than the AVA approach). As a reference, a hypothetical type-level oracle that always redirects the image to the correct type-specific classifier would result in 0.738 accuracy on the MMY level.

By replacing the CCE loss with BCE at the type-level classification, the network achieves the best result of 0.991 training set accuracy, and 0.934 test accuracy. Improving the accuracy of this first level classifier in turn improves the MMY-level accuracy because more images are forwarded to the correct classifiers and have the chance of being correctly labeled. In fact, the overall cascade accuracy with this newly trained car type classifier improves to 0.701, reaching the best result out of the presented methods. This is shown in the third column of Table 3. Such behavior is probably caused by the fact that the BCE loss does not penalize correct predictions with scores close to the ones of the other classes like the cross-entropy function does. As a further experiment, then, we replaced the loss function at the level of type-specific classifiers. This lead to a significant drop in performance (below 0.6), coherently with the findings of the AVA experiment.

The **Hierarchical multilabel (HML)** approach aims at simplifying the Two-step cascade by performing the same operations with a single classifier. To do so, it predicts simultaneously both the car type and the car MMY labels.The corresponding neural network reached 0.920 car type accuracy and 0.655 car MMY accuracy on the test, as shown in the last column of Table 3, and respectively 0.974 and 0.879 accuracy on the training set. It can be noticed that this solution achieves inferior MMY accuracy than the previously discussed two approaches with the same CCE loss (respectively 0.033 less than AVA and 0.025 less than 2SC), but at the same time it achieves a slightly higher car type accuracy (0.013 better).

Following the positive results of partial BCE loss application 2SC, we modified the HML solution to simultaneously train the car type classifier with BCE, and the MMY classifiers with CCE. The loss values are then summed together and backpropagated. However, the corresponding network only achieves 0.856 type accuracy and 0.624 MMY accuracy on the test set. Compared to the cross-entropy trained version, accuracy results on both levels are lower. A possible explanation could be found in the necessity to introduce weights when combining the two loss functions. This investigation is, however, left for future works.

**Figure 7:**
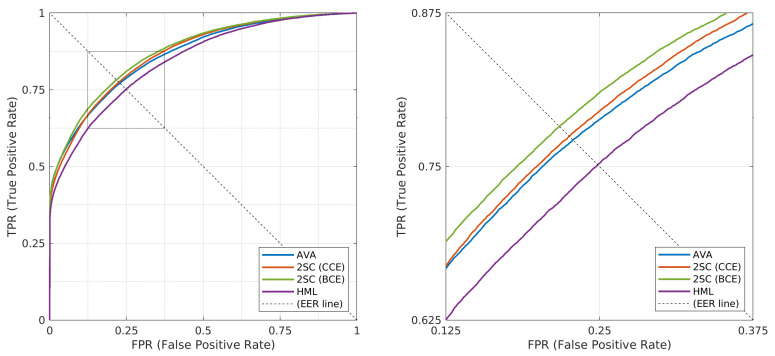
Macro-average ROC curves for different classification approaches on our revisited CompCars dataset at the finest level of classification (make–model–year). The Equal Error Rate line (EER) is also visualized.

In Figure 7, we report the Receiver Operating Characteristic (ROC) curves for the presented car classification approaches, focusing on the finest level of make–model–year. Since this is a multiclass problem, we adopted a macro-average strategy for each approach: a different ROC curve is calculated for every MMY class against all others, and the resulting curves are eventually averaged. The statistics related to the four ROC curves are also reported in Table 4, including Equal Error Rate (EER) reported by its True Positive Rate (TPR) and Area Under Curve (AUC). This analysis confirms the superiority of Two-step cascade with Binary Cross-Entropy with respect to the other presented approaches. Differently from the observations based on accuracy, then, the Two-step cascade with Categorical Cross-Entropy appears slightly better than All-vs.-all, suggesting that in fact the two methods should be considered globally comparable in performance. We make all the corresponding data available along with our revisited annotations, in order to allow other researchers to reproduce our same statistics, and to potentially compute other metrics as well.

#### Impact of the Rejection Class

**Figure 8:**
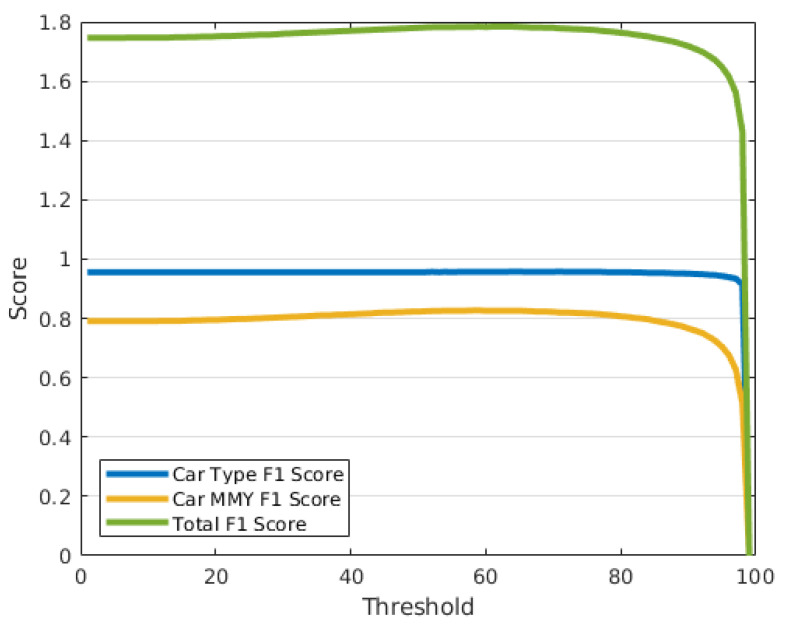
Chart of the singles and total F1 scores obtained with different threshold levels. The tested integer threshold values ranges from 0 to 100 with step 1. The highest value F1 score is 1.784 and occurs with threshold value 59.

In order to handle images belonging to an unknown class, we considered the definition of a rejection class based on thresholding the prediction confidence. To this extent, we computed the F1 score on the CompCars dataset for 100 different steps of confidence. The resulting F1 curves are shown in Figure 8 for the type label, MMY label, and a sum of the resulting F1 values (Total F1).

The best threshold value for total F1 is 59%, corresponding to 1.784. With this threshold, only 1.7% of the total images are rejected for the type classification, while there is a 20.2% rejection for the MMY classification. Out of this 20.2%, 6.2% are correct classifications with low confidence and the rest are incorrect predictions with low confidence. The 2% rejection for the car type label is for correct classifications with low confidence.

In order to perform a comparison with an external source of data, an interesting and convenient dataset for this task is the BMW-10 subset from Stanford Cars. It is composed of 512 images from 10 different models of BWM sedans (type 3), and none of these models is present in the CompCars dataset. On the BMW-10 dataset, our classification network achieves 0.829 accuracy on the car type prediction, rejecting a 2% of the images because of low confidence value (even though those are actually correct) and 3.2% of the incorrect predictions. For the MMY label, the classification accuracy cannot be evaluated; however, 78.3% of the instances are correctly rejected.

## 6. Conclusions

Recognizing cars in a digital image up to the finest detail of year-specific release can have different applications, ranging from security to advertisement. Multiple datasets have been published for this purpose, each with their own characteristics, and the CompCars dataset was found to strike a good balance between cardinality and labeling detail. We described a process that led us to revisit and enhance the annotations of this dataset with additional pieces of information, which we made available for public download. We then trained and tested different solutions for car classification, two of which take full advantage of the hierarchical nature of the annotations. A rejection-class mechanism was also implemented to address the limitations of a close-set classification setup. The best results in terms of performance at both the coarsest and the finest level of classification were obtained with a two-step cascade of classifiers: a first one that predicts the car type, trained with a binary cross-entropy loss, redirecting the subsequent processing to a second type-specific classifier, designed to predict the car make, model, and year simultaneously. Another interesting solution was the definition of a hierarchical multilabel classifier that predicts both levels of the hierarchy in a single run. This multilabel approach, which performed well for the highest level of hierarchy, could be further developed in the future by resorting to level-specific weights during the training phase. More in general, future developments might consider exploiting the dataset hierarchy at intermediate levels as well, in terms of car make and model.

## Figures and Tables

**Table 1:** Summary of the dataset characteristics for car classification. Figures marked with * indicate that the information is only available for part of the dataset.

Dataset	CompCars [13]	Stanford [14]	VMMR [15]
Total images	136,725	16,185	291,752
Train images	16,016 *	8144	N/A
Test images	14,939 *	8041	N/A
Type classes	12 *	N/A	N/A
Make classes	163	N/A	N/A
Model classes	1716	N/A	N/A
Year classes	4455	196	9170

**Table 2:** Characteristics comparison between different approaches for car classification. The first group is described in Section 4: All-vs.-all (AVA), Two-step cascade (2SC), and Hierarchical multilabel (HML). The second group is described in Section 2: Channel Max Pooling (CMP), Attention Branch Network (ABN), and the method by Fomin et al.

Approach	Exploits Hierarchy	Car Level Prediction	Single Input	Number of Classifiers	Backbone Architecture
AVA (ours)	✘	MMY	✔	1	ResNet-50
2SC (ours)	✔	Type → MMY	✔	1 + 12	ResNet-50
HML (ours)	✔	Type → MMY	✔	1	ResNet-50
CMP [18]	✘	Model	✔	1	DenseNet-161, VGG16, ResNet-152
ABN [22]	✘	Model	✔	1	VGG16, ResNet-101
Fomin et al. [23]	✔	Orientation → Make	✘	1 + 5	Inception-ResNet-v3

**Table 3:** Classification accuracy on our revisited CompCars dataset using different approaches: All-vs.-all (AVA), Two-step cascade (2SC), and Hierarchical multilabel (HML). The two-step cascade is realized with two variants, using either categorical (CCE) or binary (BCE) cross-entropy at the type-level classification. Best results per line are highlighted in boldface.

Classification Approach	AVA	2SC (CCE)	2SC (BCE)	HML
**Hierarchy Level**	**Classification Accuracy**			
Type	0.907	0.907	**0.934**	0.920
MMY (Make–Model–Year)	0.687	0.680	**0.701**	0.654
MMY for type 1: MPV	0.696	0.684	**0.722**	0.622
MMY for type 2: SUV	0.707	0.716	**0.728**	0.684
MMY for type 3: Sedan	**0.689**	0.668	0.671	0.661
MMY for type 4: Hatchback	**0.711**	0.637	0.694	0.666
MMY for type 5: Minibus	0.613	**0.681**	0.679	0.562
MMY for type 6: Fastback	0.691	0.663	**0.700**	0.663
MMY for type 7: Estate	0.679	0.692	**0.772**	0.553
MMY for type 8: Pickup	0.613	0.715	**0.727**	0.684
MMY for type 9: Hardtop	0.699	**0.754**	0.743	0.641
MMY for type 10: Sports	0.639	0.676	**0.709**	0.631
MMY for type 11: Crossover	0.706	0.735	**0.755**	0.723
MMY for type 12: Convertible	0.680	0.725	**0.745**	0.639

**Table 4:** ROC curves statistics of different classification approaches on our revisited CompCars dataset: All-vs.-all (AVA), Two-step cascade (2SC) with two losses, and Hierarchical multilabel (HML).

Classification Approach	AVA	2SC (CCE)	2SC (BCE)	HML
ROC Statistic				
Equal Error Rate (EER)	0.7715	0.7746	0.7826	0.7512
Area Under Curve (AUC)	0.8635	0.8670	0.8745	0.8439

## Data Availability

Publicly available datasets were analyzed in this study. This data can be found here: http://www.ivl.disco.unimib.it/activities/hierarchical-car-classification/.

## Abbreviations

The following abbreviations are used in this manuscript:

2SC	Two-Step Cascade
ABN	Attention Branch Network
AUC	Area Under Curve
AVA	All-Vs.-All
BCE	Binary Cross-Entropy
CCE	Categorical Cross-Entropy
CMP	Channel Max Pooling
CNN	Convolutional Neural Network
FN	False Negatives
FP	False Positives
GDPR	General Data Protection Regulation
HML	Hierarchical MultiLabel
mAP	mean Average Precision
MMY	Make–Model–Year
MPV	Multi-Purpose Vehicle
R-CNN	Regions with CNN features
ROC	Receiver Operating Characteristic
SUV	Sport Utility Vehicle
TN	True Negatives
TP	True Positives
VGG	Visual Geometry Group
VMMR	Vehicle Make and Model Recognition
VOC	Visual Object Classes
